# Identification of Potent Natural Resource Small Molecule Inhibitor to Control *Vibrio cholera* by Targeting Its Outer Membrane Protein U: An In Silico Approach

**DOI:** 10.3390/molecules26216517

**Published:** 2021-10-28

**Authors:** Abdul Rahaman, Abdulraheem Ali Almalki, Misbahuddin M. Rafeeq, Omar Akhtar, Farah Anjum, Mutaib M. Mashraqi, Ziaullah M. Sain, Ahmad Alzamami, Varish Ahmad, Xin-An Zeng, Qazi Mohammad Sajid Jamal

**Affiliations:** 1 College of Food Science and Engineering, Foshan University, Foshan 528231, China; rahaman_knabdul@ymail.com; 2 School of Food Science and Engineering, South China University of Technology, Guangzhou 510641, China; 3 Overseas Expertise Introduction Centre for Discipline Innovation of Food Nutrition and Human Health (111 Centre), Guangzhou 510641, China; 4 Department of Clinical Laboratory Sciences, College of Applied Medical Sciences, Taif University, P.O. Box Number 11099, Taif 21944, Saudi Arabia; almalki@tu.edu.sa (A.A.A.); farahanjum@tu.edu.sa (F.A.); 5 Department of Pharmacology, Faculty of Medicine, Rabigh, King Abdulaziz University, Jeddah 21589, Saudi Arabia; marafeeq@kau.edu.sa; 6 Department of Medicine, Tbilisi State Medical University, Tbilisi 0177, Georgia; a.omar2k22@gmail.com; 7 Department of Clinical Laboratory Sciences, College of Applied Medical Sciences, Najran University, Najran 61441, Saudi Arabia; mmmashraqi@nu.edu.sa; 8 Department of Microbiology, Faculty of Medicine, Rabigh, King Abduaziz University, Jeddah 21589, Saudi Arabia; zsain@kau.edu.sa; 9 Clinical Laboratory Science Department, College of Applied Medical Science, Shaqra University, Shaqra 11961, Saudi Arabia; aalzamami@su.edu.sa; 10 Health Information Technology Department, Faculty of Applied Studies, King Abdulaziz University, Jeddah 21589, Saudi Arabia; vaahmad@kau.edu.sa; 11 Department of Health Informatics, College of Public Health and Health Informatics, Qassim University, Al Bukayriyah 52741, Saudi Arabia

**Keywords:** *Vibrio cholerae*, cholera, OmpU, natural compounds, molecular dynamics, vHTS

## Abstract

*Vibrio cholerae* causes the diarrheal disease cholera which affects millions of people globally. The outer membrane protein U (OmpU) is the outer membrane protein that is most prevalent in *V. cholerae* and has already been recognized as a critical component of pathogenicity involved in host cell contact and as being necessary for the survival of pathogenic *V. cholerae* in the host body. Computational approaches were used in this study to screen a total of 37,709 natural compounds from the traditional Chinese medicine (TCM) database against the active site of OmpU. Following a sequential screening of the TCM database, we report three lead compounds—ZINC06494587, ZINC85510056, and ZINC95910434—that bind strongly to OmpU, with binding affinity values of −8.92, −8.12, and −8.78 kcal/mol, which were higher than the control ligand (−7.0 kcal/mol). To optimize the interaction, several 100 ns molecular dynamics simulations were performed, and the resulting complexes were shown to be stable in their vicinity. Additionally, these compounds were predicted to have good drug-like properties based on physicochemical properties and ADMET assessments. This study suggests that further research be conducted on these compounds to determine their potential use as cholera disease treatment.

## 1. Introduction

Cholera is a severe diarrheal infection caused by consuming food or water contaminated with the bacteria *Vibrio cholerae*. It remains a global public health problem, as well as a manifestation of injustice and a lack of social development. The global prevalence of cholera disease has risen considerably in recent years. It is estimated that cholera causes about 1.3 to 4.0 million infections and 21,000 to 143,000 fatalities worldwide each year [1]. Numerous virulence factors, including cholera toxin, toxin-co-regulated pilus, and a critical colonization factor, contribute significantly to the ability of bacteria to cause infection [2].

*V. cholerae* is a Gram-negative bacteria that causes severe diarrhea after colonizing the small intestine and inducing virulence factors [3]. *V. cholerae*, as with the majority of Gram-negative bacteria, produces outer membrane vesicles during growth, which entrap the periplasm and transmit bacterial DNA [4]. It produces a diverse set of outer membrane proteins (OMPs) with varying component molecular masses. Among these OMPs, OmpU and OmpT have been thoroughly investigated in terms of virulence, adhesion, and host colonization [5]. ToxR and/or ToxS, which control and express genes encoding porins such as OmpO and OmpT, regulate a large range of *V. cholerae* virulence genes [6,7]. If *V. cholera* is cultured on salt-free media, OmpU accounts for around 60% of its OMPs [8]. OmpU creates nonspecific barrel channels that allow hydrophilic molecules to freely diffuse through the outer membrane. OmpU possesses porin properties and has been shown to provide bile and antibacterial peptide resistance to the bacterium [9]. 

Natural products (NPs), which have greater structural variety than synthesized molecules, have traditionally been the primary sources of bioactive substances and continue to play a vital role in novel drug development [10]. Virtual screening approaches have revolutionized the identification of novel compounds with particular bioactivity by evaluating vast structural libraries against a target protein, reducing the cost, infrastructure, and time required to identify new chemo-structures [11]. These approaches employ a series of processes aimed at screening and choosing molecules with acceptable physicochemical, pharmacokinetic, and pharmacodynamic qualities while eliminating those that do not fit the desirable properties [12]. This study aimed to find new potential leads from the traditional Chinese medicine (TCM) database using in silico approaches that could be used as OmpU inhibitors to fight cholera infection.

## 2. Material and Methods

### 2.1. Protein Preparation and Binding Site Identification

The 3D structure of OmpU (PDB ID: 5ONU) was retrieved from the protein data bank. It is a homo 3-mer trimeric OmpU structure [13]. The monomer structure was selected for structural analysis, pocket identification, and the virtual screening process. UCSF Chimera (version 1.15) was employed for the structural optimization of the monomer protein including energy minimization [14]. Further, the binding site was predicted by the CASTp server [15] and COACH [16] in order to analyze the active site of the monomer 3D structure of the target protein, as well as the active-site amino acid residues.

### 2.2. Compound Library Preparation, Virtual Screening, and Molecular Docking

The TCM database is one of the world’s largest non-commercial traditional Chinese medicine compound databases [17]. The TCM database was acquired in ‘sdf’ format from the ZINC database and refined using the ligand preparation tool in Discover Studio 2020. The prepared library of compounds was converted into ‘pdbqt’ format to perform virtual screening. AutoDock Vina (version 1.1.2) [18] and AutoDock (version 4.2.5.1) [19] were employed for virtual screening and in-depth molecular docking analysis. 

### 2.3. Physiochemical Properties and ADMET Prediction

The physiochemical properties and ADMET predictions were assessed for the top screened compounds by employing Datawarrior tools [20] and the SwissADME web server [21]. Compounds that fulfilled the chosen criteria, particularly Lipinski’s rule, were selected for further investigation.

### 2.4. Molecular Dynamic (MD) Simulation

MD simulations were conducted on free OmpU, the OmpU-control compound, OmpU-ZINC95910434, and OmpU-ZINC06494587 at 300 K using GROMACS 5.1.2 [22] by employing the GROMOS96 43a1 force field [23]. The PRODRG server was employed to produce the compounds’ topology and force field parameters [24]. 

Using the gmx editconf module for establishing boundary conditions and the gmx solvate module for solvation, the free OmpU, OmpU-control, OmpU-ZINC95910434, and OmpU-ZINC06494587 were soaked in a ‘cubic box’ of water molecules with an initial diameter of 8 nm. Charges on free OmpU and its complexes were neutralized by utilizing the gmx genion module to introduce Na^+^ and Cl^-^ ions to preserve neutrality and maintain a physiological concentration (0.15 M). The system was then minimized using 1500 steps of ‘steepest descent’, and the temperature of all systems was increased from 0 to 300 K throughout their equilibration time (100 ps) while maintaining a constant volume and periodic boundary conditions. 

Equilibration took place in two stages: NVT ensemble and NPT ensemble. The C^α^ backbone atoms of the original structures were restrained with all other atoms allowed to move freely in both NVT and NPT. Then, MD was performed at a time scale of 100 ns at 300 K. The resulting trajectories were investigated using GROMACS analysis modules. PyMOL and VMD were used to create all graphical representations of the 3D models [25]. 

## 3. Results and Discussion

OmpU is a virulence component of *V. cholerae* that is involved in host cell interaction and is necessary for pathogenic *V. cholerae* survival in the host body [26], making it a potential target in the management of cholera infection. Virtual screening is a computer-assisted method for discovering potential compounds that can bind to a known target molecule, and it is becoming an increasingly successful approach for finding novel inhibitors and therapeutic compounds [27]. This study screened biogenic compounds from the TCM database against the OmpU of *V. cholerae*. Among them, lead compounds ZINC06494587, ZINC85510056, and ZINC95910434 show good binding with OmpU.

**Figure 1:**
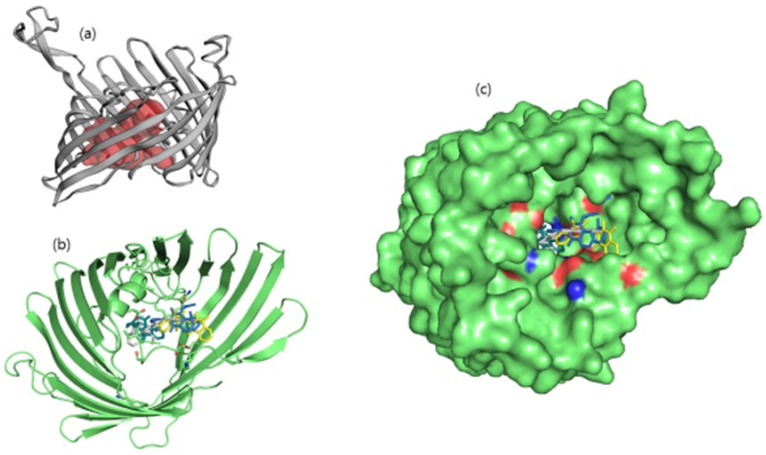
(**a**) Illustration of predicted protein pocket. (**b**,**c**) The best compounds are shown in the pocket of OmpU, as well as in a surface view depiction.

Binding pocket residues of the OmpU protein were predicted as Gly32, Asn34, Gln35, Ser36, Gly37, Asp38, Lys39, Ala40, Gly41, Glu53, Gly55, Arg76, Leu80, Val92, Phe94, Glu96, Arg116, Tyr117, Tyr119, Glu128, Thr130, Asn134, Asp135, Gly136, Ala137, Gly139, Val140, Thr142, Asp143, Asp146, Asn153, Lys158, Ala162, Asn163, Arg164, Ala170, Tyr171, Lys172, Lys181, Ala182, Ser183, and Tyr312 (Figure 1). Consistent with this, leads ZINC06494587, ZINC85510056, and ZINC95910434 were also found to bind with these residues of OmpU.

Table 1 shows the physicochemical characteristics of the top 24 screened phytochemicals as filtered by Lipinski’s rule. The compounds in bold are the selected compounds for the detailed interaction analysis in this study. Our analysis indicated that the natural compounds we evaluated exhibited significant drug-like characteristics across a range of parameters (Table 1 and Table 2).

**Figure 2:**
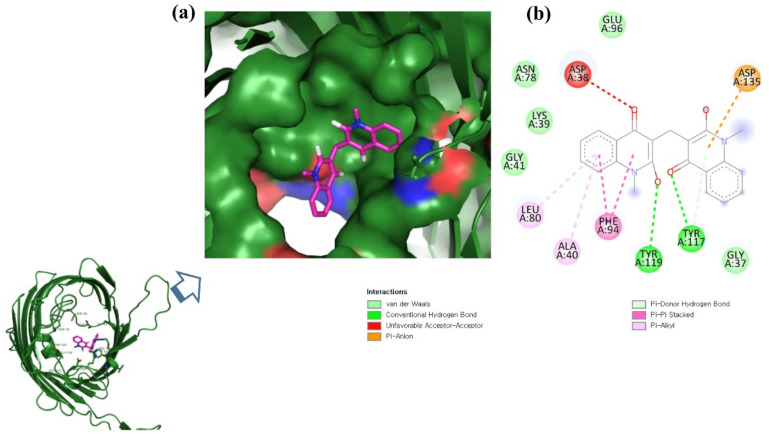
Visualization of ZINC06494587 in the binding pocket of OmpU (**a**); 2D view of residues of OmpU interacting with ZINC06494587 (**b**).

**Figure 3:**
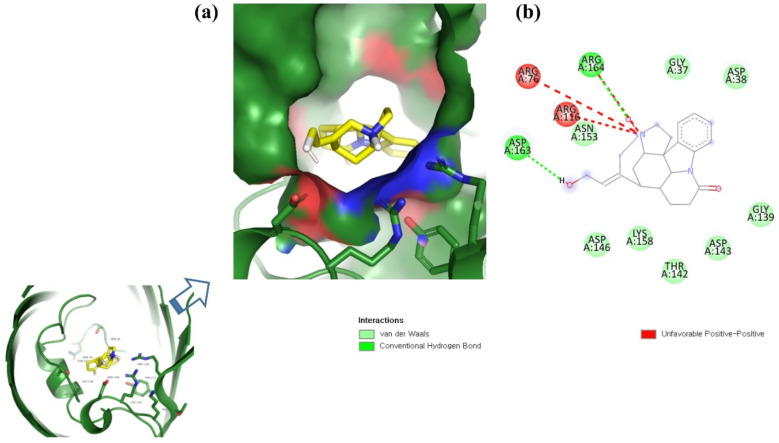
Visualization of ZINC85510056 in the binding pocket of OmpU (**a**); 2D view of residues of OmpU interacting with ZINC85510056 (**b**).

**Figure 4:**
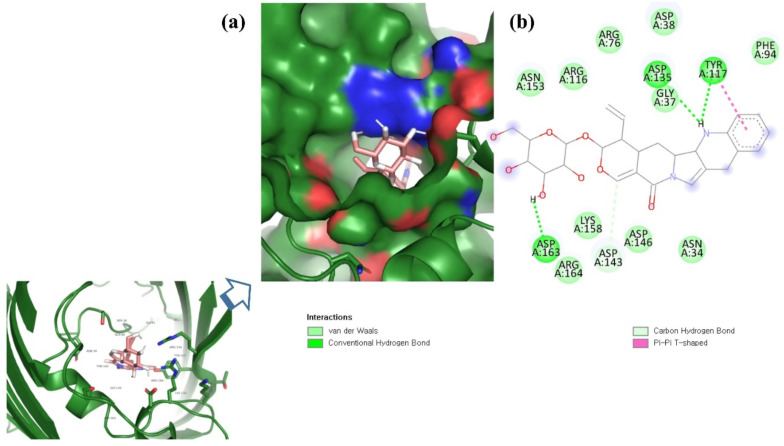
Visualization of ZINC95910434 in the binding pocket of OmpU (**a**); 2D view of residues of OmpU interacting with ZINC95910434 (**b**).

ZINC06494587 interacted with the Glu96, Asp135, Gly37, Tyr117, Tyr119, Phe94, Ala40, Leu80, Gly41, Lys39, Asn78, and Asp38 residues of OmpU. The Gly41, Lys39, Asn78, Glu96, and Gly37 residues were involved in van der Waals interactions with ZINC06494587 (Figure 2). The Arg164, Gly37, Asp38, Gly139, Asp143, Thr142, Lys158, Asp146, Asp163, Asn153, Arg116, and Arg76 residues of OmpU were found to interact with ZINC85510056. The Gly37, Asp38, Gly139, Asp143, Thr142, Lys158, Asp146, and Asn153 residues engaged in van der Waals interactions with ZINC85510056 (Figure 3). Further, ZINC95910434 interacted with the Asn153, Arg116, Arg76, Asp38, Asp135, Gly37, Tyr117, Phe94, Asn34, Asp146, Asp143, Lys158, Arg164, and Asp163 residues of OmpU. ZINC95910434 exhibited van der Waals interactions with the Asn153, Arg116, Arg76, Asp38, Gly37, Phe94, Asn34, Asp146, Lys158, and Arg164 residues of OmpU (Figure 4). The binding affinity values for lead compounds ZINC06494587, ZINC85510056, and ZINC95910434 with OmpU were found to be −8.92, −8.12, and −8.78 kcal/mol, respectively (Table 3).

The H-bond is one of the most important interactions between an inhibitor and a protein, and it exhibits a critical role in the stability of ‘inhibitor–protein’ complexes [28]. ZINC06494587 established a H-bond with the Tyr117 and Tyr119 residues of OmpU (Figure 2), while the Arg164 and Asp163 residues were involved in a H-bond with ZINC85510056 (Figure 3). Further, ZINC06494587 established a H-bond with the Tyr117, Asp135, and Asp163 residues of OmpU (Figure 4).

**Figure 5:**
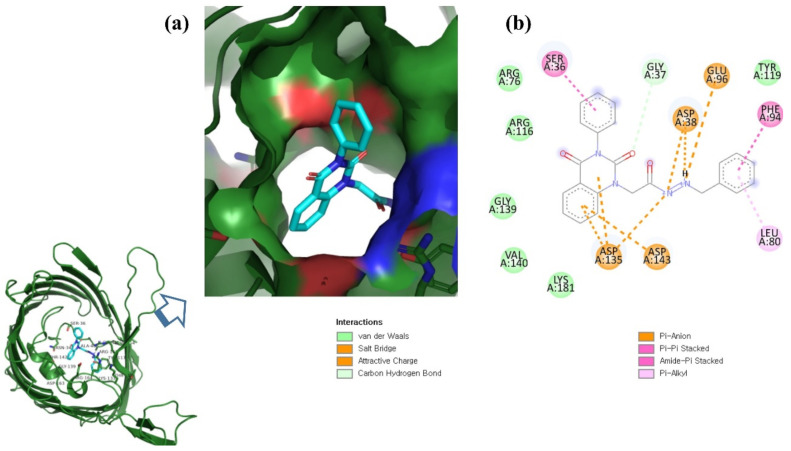
Visualization of control compound in the binding pocket of OmpU (**a**); 2D view of residues of OmpU interacting with the control compound (**b**).

This study used N′-[2-(2,4-dioxo-3-phenyl-3,4-dihydro-2H-quinazolin-1-yl)-acetyl]-hydrazide as a control compound due to its previously reported strong interaction with the OmpU protein, with an MIC value of 10 μg/mL [29]. The control compound was found to interact with the Arg116, Arg76, Ser36, Gly37, Glu96, Asp38, Tyr119, Phe94, Leu80, Asp143, Asp135, Lys181, Val140, and Gly139 residues of OmpU (Figure 5). Lead compounds ZINC06494587, ZINC85510056, and ZINC95910434 were also found to interact with these residues of OmpU (Figure 2, Figure 3 and Figure 4). The binding affinity value for the control compound with OmpU was noted to be −7.0 kcal/mol (Table 3).

NPs exhibit a vital role in drug discovery [30,31]. More than half of the FDA-approved drugs are NPs or NP derivatives [32]. In addition, natural compounds have a high selectivity for cellular targets [33]. NPs with biological activity can function as selective ligands for disease-related targets, affecting disease-associated pathways and shifting the biological network from disease to health status [34]. 

The goal of inhibitor–protein docking is to anticipate the most likely binding mode(s) of an inhibitor with a known 3D structure of the protein. A high binding (or more negative) affinity value obtained in docking indicates an efficient interaction between ligand–protein complexes [35]. Accordingly, ZINC06494587, ZINC85510056, and ZINC95910434 showed strong binding with OmpU, with higher binding affinity values than the control compound, suggesting that these leads can be used as OmpU inhibitors to fight cholera disease.

**Figure 6:**
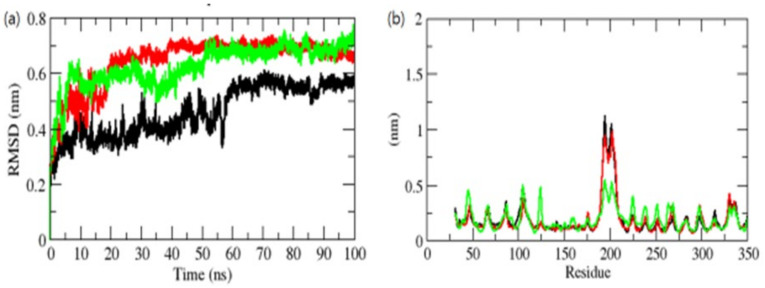
RMSD (**a**) and RMSF (**b**) of complexes. The OmpU-control compound, OmpU-ZINC95910434, and OmpU-ZINC06494587 are shown in black, red, and green color, respectively.

The binding of a chemical to a protein’s catalytic domain can cause significant conformational changes in the protein [36]. The root mean square deviation (RMSD) is a critical fundamental characteristic for determining if a protein is stable and conforms to its experimental structure [37]. The average values of RMSD for the OmpU-control compound, OmpU-ZINC95910434, and OmpU-ZINC06494587 were found to be 0.42 nm, 0.61 nm, and 0.50 nm, respectively. According to the RMSD plot, the binding of the control and ZINC06494587 stabilized OmpU and resulted in fewer structural aberrations from its natural conformation (Figure 6a). 

In the instance of the ZINC95910434 complex binding to the OmpU active pocket (AP), early modest variations were seen for the first 10–20 ns of the MD trajectories, after which a significant RMSD value was reached. During the 100 ns MD simulations, the orientation of the control and ZINC06494587 in the AP of OmpU had the lowest RMSD values and was shown to be equilibrated. The control and ZINC95910434 showed incessant fluctuations in the AP of OmpU, probably due to the diverse orientations; the main region of fluctuation was observed between the 170 and 220 residues (Figure 6b). The vibrations surrounding the equilibrium are not random but rather rely on the flexibility of the local structure. The root mean square fluctuation (RMSF) of OmpU upon the binding of selected compounds was displayed as a function of residue numbers in order to compute the average fluctuation of all residues during the simulation. The RMSF plot revealed that residual variations exist in OmpU at various locations of the protein structure. These residual fluctuations were shown to be reduced by the binding of ZINC06494587 and increased by the binding of the control and ZINC95910434.

**Figure 7:**
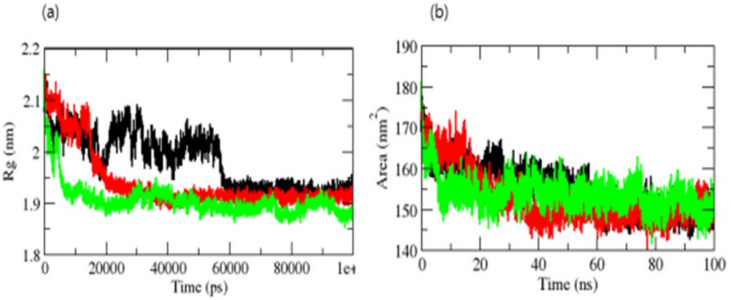
Rg (**a**) and SASA of complexes (**b**). The OmpU-control compound, OmpU-ZINC95910434, and OmpU-ZINC06494587 are shown in black, red, and green color, respectively.

The radius of gyration (Rg) is a metric related to a protein’s tertiary structure volume that is used to gain insight into the protein’s stability in a biological system. A protein should have a larger radius of gyration due to its less dense packing. The average *R_g_* values for the OmpU-control compound, OmpU-ZINC95910434, and OmpU-ZINC06494587 were found to be 2.0 nm, 1.90 nm, and 1.81 nm, respectively. The Rg plot indicated that OmpU achieved tighter packing in OmpU-ZINC95910434 and OmpU-ZINC06494587 (Figure 7a).

The surface area of a protein that interacts with its solvent molecules is referred to as the solvent-accessible surface area (SASA) [38]. Average SASA values for the OmpU-control, OmpU-ZINC95910434, and OmpU-ZINC06494587 complexes were observed throughout the simulations (Figure 7b). The average SASA values for the OmpU-control, OmpU-ZINC95910434, and OmpU-ZINC06494587 complexes were found to be 155.12 nm^2^, 150.81 nm^2^, and 156.23 nm^2^, respectively. 

**Figure 8:**
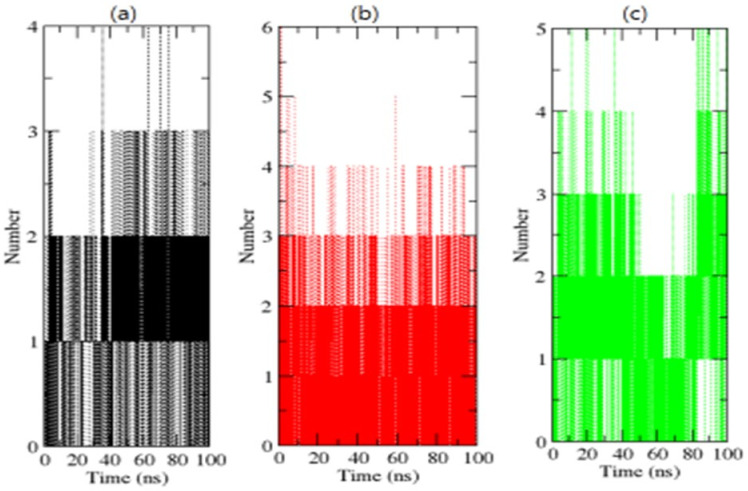
H-bond analysis of complexes. The OmpU-control compound, OmpU-ZINC95910434, and OmpU-ZINC06494587 are shown in black, red, and green color, respectively.

H-bonding amongst proteins and chemicals enables interaction directionality and specificity, which is a crucial element of molecular recognition [39]. To confirm the stability of complexes, the H-bonds paired within 0.35 nm between the OmpU-control compound, OmpU-ZINC95910434, and OmpU-ZINC06494587 were calculated during the 100 ns simulations. ZINC95910434 and ZINC06494587 were found to strongly bind to the AP of OmpU with three–four H-bonds, while the control compound bound to the AP of OmpU with one–two H-bonds, with the least fluctuations (Figure 8). 

**Figure 9:**
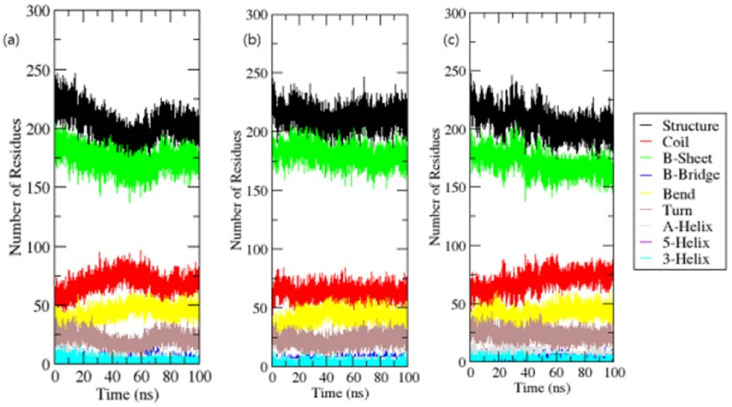
Secondary structure elements.

For each time step, secondary structural assignments in proteins such as helix, sheet, and turn were fragmented into particular residues. In contrast to OmpU, the average number of residues involved in secondary structure formation in complexes was somewhat reduced due to an increase in the proportion of coils and a decrease in sheets. In the instance of OmpU-ZINC06494587, the proportions of sheets and helices were found to be significantly lower (Figure 9). 

**Figure 10:**
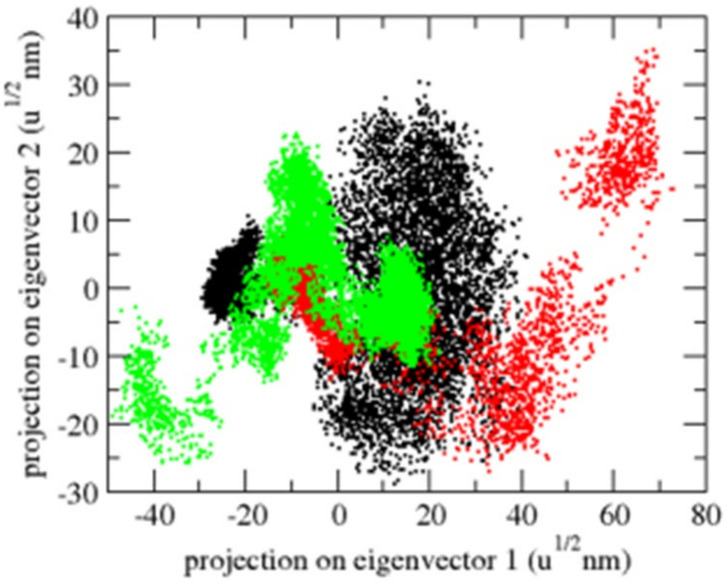
The 2D projection of complexes. The OmpU-control compound, OmpU-ZINC95910434, and OmpU-ZINC06494587 are shown in black, red, and green color, respectively.

Principal component analysis (PCA) displays a protein’s overall expansion throughout several simulations [40]. The dynamics of OmpU were computed using the gmx covar module in relation to the backbone for PCA. PCA detects a protein’s large-scale average motion, revealing the structures behind atomic fluctuations [41]. The sum of the eigenvalues is a measurement of the system’s overall motility. It may be used to compare a protein’s flexibility under various circumstances [42]. The OmpU-control compound and OmpU-ZINC06494587 complexes exhibited overlap in the 2D projections of trajectories on eigenvectors. The findings further show that a variation in atom positions is caused by complexes binding to OmpU (Figure 10).

**Figure 11:**
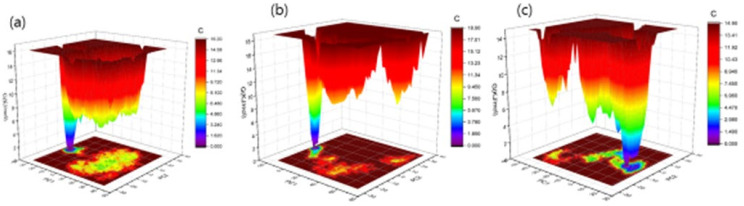
The Gibbs free energy landscape of complexes. The OmpU-control compound (**a**), OmpU-ZINC95910434 (**b**), and OmpU-ZINC06494587 (**c**).

The Gibbs free energy landscape was also modeled, using Gromacs analysis commands, and utilizing projections of their respective first (PC1) and second (PC2) eigenvectors. The corresponding contour map of the Gibbs free energy represents less energy with a darker blue shade. Due to the binding of complexes to OmpU, the global minima of OmpU change during the simulations. OmpU-control and OmpU-ZINC95910434 showed a similar projection, and OmpU-ZINC06494587 showed two global minima; therefore, the ZINC06494587 compound formed a more stable complex in the vicinity of the protein (Figure 11). 

## 4. Conclusions

OmpU is a crucial virulence component of *V. cholerae* that is engaged in host cell interaction and is necessary for the survival of pathogenic *V. cholerae* in the host body. This paper described the computational screening of TCM biogenic compounds against the OmpU protein to identify possible OmpU inhibitors able to combat cholera infection. Lead compounds ZINC06494587, ZINC85510056, and ZINC95910434 bind strongly to OmpU, with ZINC06494587 and ZINC95910434 showing stability, as revealed by MD simulations. This study suggests that further research be conducted on these compounds to optimize their potential use as cholera disease treatment. 

## Figures and Tables

**Table 1:** Physicochemical properties of top 24 screened phytochemicals and control compound filtered by Lipinski’s rule.

S. No	Molecule Name	TORSDO	Mol. Weight	cLogP	cLogS	H-Acceptors	H-Donors	Drug Score
1	Control	6	400.437	2.1389	−5.289	7	2	0.610857
2	ZINC00119434	0	335.426	1.5935	−3.554	4	1	0.831709
3	ZINC01716562	0	364.444	1.2222	−3.252	5	0	0.839942
4	ZINC06494587	2	362.384	1.8526	−4.474	6	0	0.726505
5	ZINC14588133	0	424.499	3.2063	−4.481	5	0	0.58483
6	ZINC14611877	0	381.45	1.1613	−3.401	6	1	0.817261
7	ZINC15121726	0	318.375	0.9845	−3.493	4	1	0.845727
8	ZINC15211799	0	313.359	3.1159	−5.781	4	1	0.577681
9	ZINC33967683	0	303.364	2.7404	−3.55	4	1	0.82254
10	ZINC34172946	0	264.283	1.9186	−3.766	4	2	0.812478
11	ZINC59587520	2	352.433	0.9499	−1.303	5	1	0.564557
12	ZINC59587626	0	365.452	1.2222	−3.252	5	1	0.839179
13	ZINC70451098	3	446.458	1.0959	−4.58	8	2	0.273407
14	ZINC70454552	0	350.417	0.6201	−1.385	5	0	0.701361
15	ZINC70455615	0	350.417	0.6201	−1.385	5	0	0.701361
16	ZINC85506732	4	359.401	−0.7109	−2.716	7	1	0.416303
17	ZINC85508615	3	345.418	0.4553	−2.903	6	2	0.415682
18	ZINC85510056	2	352.433	0.9499	−1.303	5	1	0.564557
19	ZINC85531660	3	393.505	2.4712	−3.935	5	1	0.743661
20	ZINC85532094	0	289.337	2.2665	−3.576	4	1	0.835381
21	ZINC85916780	2	447.446	2.2223	−4.676	9	2	0.379896
22	ZINC95910132	8	377.732	−1.1595	−1.558	10	5	0.227975
23	ZINC95910434	8	498.53	−0.162	−3.012	10	5	0.390707
24	ZINC95910792	0	320.391	1.2599	−3.721	4	1	0.828277
25	ZINC95912493	2	350.417	0.8454	−1.039	5	1	0.685761

**Table 2:** ADMET prediction of control and top 24 screened phytochemicals.

S.NO.	Molecule Name	Mutagenic	Tumorigenic	Irritant	BBB Permeant	GI Absorption	TPSA	CYP2D6 Inhibitor	Log S	PAINS Alerts
1	Control	x	x	x	N	H	85.13	No	−4.42	0
2	ZINC00119434	x	x	x	Y	H	41.57	Y	−2.43	0
3	ZINC01716562	x	x	x	Y	H	49.85	Y	−0.89	0
4	ZINC06494587	x	x	x	N	H	74.76	N	−3.32	0
5	ZINC14588133	x	x	x	Y	H	53.23	N	−3.88	0
6	ZINC14611877	x	x	x	N	H	67.87	Y	−2.35	0
7	ZINC15121726	x	x	x	Y	H	49.41	Y	−2.46	0
8	ZINC15211799	x	x	x	Y	H	42.2	Y	−4.48	0
9	ZINC33967683	x	x	x	Y	H	39.34	Y	−3.59	1
10	ZINC34172946	x	x	x	Y	H	58.2	Y	−2.22	0
11	ZINC59587520	x	x	x	N	H	69.97	Y	−1.51	0
12	ZINC59587626	x	x	x	Y	H	58.64	N	−1.07	0
13	ZINC70451098	x	x	H	N	H	101.09	Y	−3.23	0
14	ZINC70454552	x	x	x	Y	H	58.97	Y	−2.3	0
15	ZINC70455615	x	x	x	Y	H	58.97	Y	−2.3	0
16	ZINC85506732	H	L	x	Y	H	71.61	Y	−2.3	0
17	ZINC85508615	H	L	x	N	H	85.02	N	−2.32	0
18	ZINC85510056	x	x	x	N	H	69.97	N	−1.51	0
19	ZINC85531660	x	x	x	Y	H	50.8	N	−3.99	0
20	ZINC85532094	x	x	x	Y	H	46.92	N	−3.21	0
22	ZINC85916780	x	x	x	N	H	112.35	Y	−3.6	0
23	ZINC95910132	x	x	x	N	L	149.15	N	−1.7	0
24	ZINC95910434	x	x	x	N	L	140.95	N	−2.26	0
25	ZINC95910792	x	x	x	Y	H	49.41	Y	−2.57	0
26	ZINC95912493	x	x	x	Y	H	69.97	Y	−2.57	0

(H: high; L: low; N: no; Y: yes).

**Table 3:** Binding affinity values of leads with the OmpU protein.

S. No.	Compounds	2D Structure	Binding Affinity (kcal/mol)	Binding Residues
1	ZINC06494587	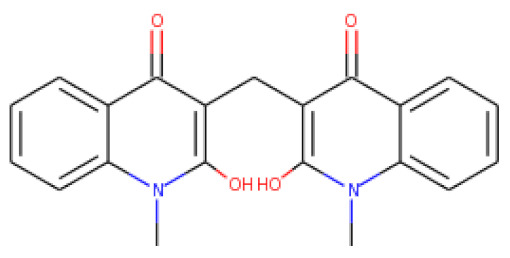	−8.92	Glu96, Asp135, Gly37, Tyr117, Tyr119, Phe94, Ala40, Leu80, Gly41, Lys39, Asn78, and Asp38
2	ZINC85510056	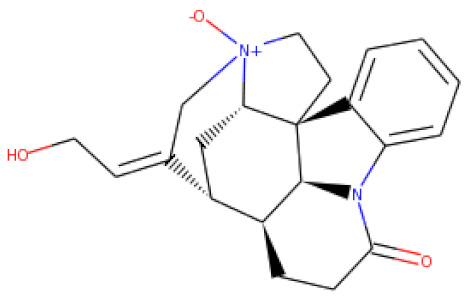	−8.12	Arg164, Gly37, Asp38, Gly139, Asp143, Thr142, Lys158, Asp146, Asp163, Asn153, Arg116, and Arg76
3	ZINC95910434	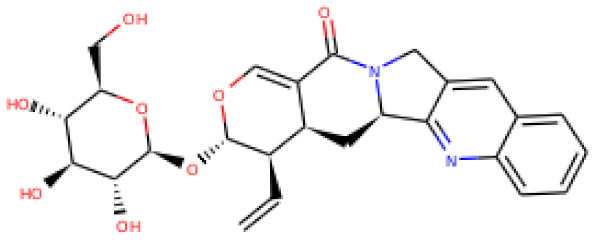	−8.78	Asn153, Arg116, Arg76, Asp38, Asp135, Gly37, Tyr117, Phe94, Asn34, Asp146, Asp143, Lys158, Arg164, and Asp163
4	Control compound *	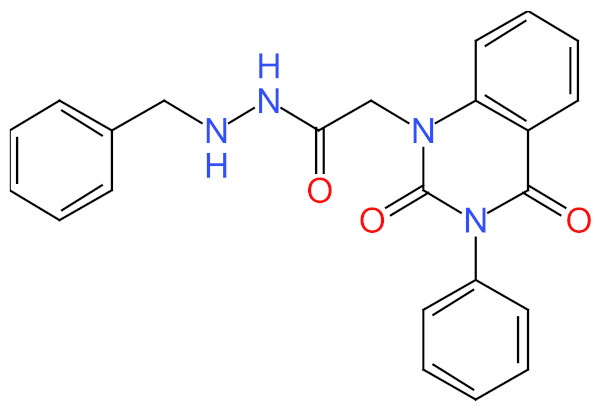	−7.0	Arg116, Arg76, Ser36, Gly37, Glu96, Asp38, Tyr119, Phe94, Leu80, Asp143, Asp135, Lys181, Val140, and Gly139

* N′-[2-(2, 4-dioxo-3-phenyl-3,4-dihydro-2H-quinazolin-1-yl)-acetyl]-hydrazide.

## Data Availability

Not applicable.
